# Association between Short Hours of Sleep and Overweight/Obesity in Mexican Adolescent Population: A School-Based Cross-Sectional Study

**DOI:** 10.3390/healthcare9080939

**Published:** 2021-07-26

**Authors:** Ana Fresan, Alma Delia Genis-Mendoza, María Lilia López-Narváez, Tania Guadalupe Gómez-Peralta, Daniela Georgina Aguilar-Velázquez, Isela Esther Juárez-Rojop, Thelma Beatriz González-Castro, Carlos Alfonso Tovilla-Zárate, Rosa Giannina Castillo-Avila, Humberto Nicolini

**Affiliations:** 1 Subdirección de Investigaciones Clínicas, Instituto Nacional de Psiquiatría Ramón de la Fuente Muñiz, Ciudad de México 14370, Mexico; a_fresan@yahoo.com.mx; 2 Instituto Nacional de Medicina Genómica, Secretaría de Salud, Ciudad de México 14610, Mexico; adgenis@inmegen.gob.mx; 3 Hospital Chiapas Nos Une Dr. Gilberto Gómez Maza, Secretaría de Salud de Chiapas, Tuxtla Gutiérrez 29045, Mexico; 4 División Académica Multidisciplinaria de Comalcalco, Universidad Juárez Autónoma de Tabasco, Comalcalco 86040, Mexico; tania1950@live.com.mx (T.G.G.-P.); gina_aguilar93@hotmail.com (D.G.A.-V.); alfonso_tovillaz@yahoo.com.mx (C.A.T.-Z.); 5 División Académica de Ciencias de la Salud, Universidad Juárez Autónoma de Tabasco, Villahermosa 86100, Mexico; iselajuarezrojop@hotmail.com (I.E.J.-R.); gianninaavila2012@hotmail.com (R.G.C.-A.); 6 División Académica Multidisciplinaria de Jalpa de Méndez, Universidad Juárez Autónoma de Tabasco, Jalpa de Méndez 86205, Mexico; thelma.glez.castro@gmail.com

**Keywords:** sleep, obesity, adolescents, women, Mexican population

## Abstract

*Background/Aim*: Obesity in adolescents is increasing; as such, the aim of this study was to determine the prevalence of obesity in Mexican adolescents and examine its possible association with hours of sleep. *Methods*: A school-based cross-sectional study was carried out. This study included 863 adolescents aged between 11 and 16 years. The prevalence of obesity was estimated using the body mass index (BMI). The duration of sleep (and other information) was assessed by a self-reported questionnaire. The Cochran–Mantel–Hansel test for categorical variables and a general linear model for continuous variables were used to evaluate the interaction effect of BMI and sex with respect to sleeping and assessed activity conditions. *Results*: It was found that 47.6% of the adolescents were overweight/obese. Men were more frequently overweight/obese than women (52.6% vs. 41.8%, *p* = 0.002). Moreover, overweight/obese adolescents were younger and spent fewer daily hours watching television (*p* < 0.05). Men practiced sports more hours per week than women (*p* = 0.04). However, women spent more daily time on the internet (*p* = 0.05), and overweight/obese adolescent women slept fewer hours than overweight/obese men and adolescents with normal weight (*p* = 0.008). *Conclusions*: The development of strategies for the prevention of overweight/obesity and the improvement of sleep duration should include a gender perspective to improve health habits in Mexican adolescents.

## 1. Introduction

Overweight and obesity are worldwide health problems; in particular, in school adolescents, overweight and obesity are rapidly increasing. It is known that, from 1990 to 2012, the prevalence increased from 4.2% to 7.0%; in 2016, almost 340 million children and adolescents were overweight or obese [1,2]. In the USA, 32% of children and adolescents were diagnosed as overweight or obese in 2007–2008 [3], whereas, in Spain, in 2012, the prevalence was 40% [4]. In Mexico, the prevalence of overweight and obesity in 2012 among the general population was 71.3% (38.8% overweight and 32.4% obese) [5], while 35% of the adolescents presented overweight and obesity [6,7,8]. In the state of Tabasco only, overweight and obesity in adolescents reached 40.7% in the same year [7].

Literature reports have indicated that several medical complications are common in children with obesity, such as orthopedic, metabolic, and cardiovascular diseases [9,10,11,12]; therefore, prevention and treatment of obesity and overweight are crucial [13]. There are many factors associated with overweight and obesity in adolescents, such as sedentary lifestyles (many hours using computers or watching television) and fast food intake [14,15,16]. Furthermore, some studies have suggested that few hours (<8 h) of sleep every night could also be associated with obesity [17,18].

In 2004, Spiegel et al. [19] reported an increase in hunger and appetite in young men associated with sleep curtailment. Then, a growing interest in the association between a few hours of sleep and obesity was attained. Since 2004, many studies have investigated the association between a few hours of sleep and obesity in adolescents [17,20,21,22]; nevertheless, the mechanism of association is not entirely known [23]. Despite the increasing reports in this area and a meta-analysis including 35,540 participants [24], nonconclusive outcomes were attained. To date, no clear association has been established between a short time of sleep and obesity in the Mexican adolescent population; consequently, the present study is required. In this work, we determined the frequency of obesity by gender in a school-based cross-sectional study and evaluated, among other variables related to sleep and physical activities, the relationship between sleep duration per night and obesity by sex, in a sample of adolescents in the southeastern area of Mexico.

## 2. Materials and Methods

This was a school-based cross-sectional study, among students of public schools in Comalcalco, Tabasco, México. We assessed students of junior high school between 11 and 16 years of age; a total of 863 adolescents were included. The schools were selected according to the geographic regions in Comalcalco city. Three schools were chosen, due to their representativeness of the municipality. In order to have a balanced geographic distribution, only 350 participants per school were included.

All parents received detailed information on the aims of the study. Only adolescents whose mother or father signed the consent form were included in the study. Neither the adolescents nor their relatives received any economical remuneration. This study was conducted following the guidelines of the Declaration of Helsinki. This study was approved by the DAMC-UJAT Ethics and Research Committee (UJAT-DAMC-2013B-004).

### 2.1. Data Collection

Personal information was collected using a self-reported structured questionnaire designed for this study. Demographics such as age and sex were included.

### 2.2. Obesity Evaluation

The anthropometric measurements of adolescents were undertaken in their school by five trained medical students attending their third year of university school. Measurement techniques of the medical students were standardized prior to the study to avoid evaluation biases. Height was measured (in meters) using stadiometers with 1 mm precision, and body weight was measured (in kilograms) using a digital bascule with 0.1 kg precision. Overweight and obesity were classified according to the International Obesity Task Force criteria (IOTF), based on body mass index measurements (BMI), with a cutoff based on BMI curves as a function of sex and age defined by the literature [25], as well as in compliance with the Mexican Official Norm NOM-008-SSA2-1993, which refers to the nutrition, growth, and development of children and adolescents.

### 2.3. Sleep Duration

To obtain information about sleep duration (including hours slept per night), a questionnaire was developed following previous procedures reported in the literature [17,26]. We included the following questions: “*How many hours do you sleep at night before a school day (i.e., Sunday, Monday, Tuesday, Wednesday, and Thursday)? How many hours do you sleep at night before days on which you do not attend school (Friday and Saturday)?*” With this information, we calculated the average hours slept per day. The average duration of sleep was calculated as reported by Mitchell et al.: (school night × 5) + weekend night × 2)/7 [17]. An additional question related to sleep was added (“*Do you normally wake up in the night to drink water?*”), as this behavior may fragment sleep continuity. This questionnaire also collected information for other variables such as the number of days and daily hours playing videogames, watching television, and using the internet. Furthermore, other activities such as reading and practicing sports (sports, gym, running) were also registered.

### 2.4. Statistical Analysis

All statistical procedures were performed using the Statistical Package for the Social Sciences (SPSS), version 21. Descriptive statistics of all variables were calculated with frequencies and percentages for categorical variables and means and standard deviations for continuous variables. The Cochran–Mantel–Haenszel test was used to examine sex differences on the categorical variables assessed between BMI groups (normal weight vs. overweight/obese) with the estimated odds ratio (95% C.I.) for three-way contingency tables (2 × 2 × 2). A generalized linear model (GLM) was performed to assess mean changes in continuous variables between men and women, as well as among BMI groups, and to test the interaction effect of sex × BMI groups. All tests were conducted at a two-sided 5% significance level.

## 3. Results

A total of 863 Mexican adolescents with a mean age of 13.0 years (*S.D.* = 1.0, range 11–16 years) were included. A similar proportion of men (46.9%, *n* = 405) and women (53.1%, *n* = 458) was found. A total sleep time of 8.5 h (*S.D*. = 1.3, range 4–14) was reported, with a high percentage of adolescents reporting sedentary activities such as watching television daily (92.1%, *n* = 795) and the use of the internet (73.8%, *n* = 637). The remaining sleep variables, as well as the sedentary and physical activities assessed, are included in Table 1.

In accordance with the previously defined cutoff points to determine BMI groups, 52.4% (*n* = 452) of the adolescents were classified into the normal weight group, while the remaining 47.6% (*n* = 411) were classified into the overweight/obese group. Overweight/obesity was more frequently reported in men (52.6%, *n* = 216) than in women (41.8%, *n* = 189; χ^2^ = 9.9, *p* = 0.002).

The comparisons between BMI groups according to sex are displayed in Table 1. According to the Breslow–Day Tarone-adjusted value of the Cochran–Mantel–Haenszel test for categorical variables and the Levene test of the GLM for continuous variables, all comparisons were homogeneous with values over 0.05. No significant differences emerged in the frequency of presentation of sleep variables, as well as sedentary or physical activities, between BMI groups according to sex. It was observed that more overweight/obese men reported reading as an activity compared to normal weight men (47.7% vs. 37.6%, χ^2^ = 4.2, *p* = 0.04).

Results of the GLM showed significant differences between BMI groups as a function of age (Pillai’s trace = 0.22, F = 9.8, *p* = 0.003), where overweight/obese adolescents were younger, as well as between sexes as a function of daily time spent on the internet (Pillai’s Trace = 0.23, F = 4.0, *p* = 0.05), where women spent more time than men, and number of days practicing physical activities (Pillai’s trace = 0.23, F = 4.2, *p* = 0.04), where men practiced physical activities (sports) more often than women. Interestingly, the only significant interaction effect of sex × BMI groups was observed in the mean hours of sleep (Pillai’s trace = 0.25, F = 4.2, *p* = 0.04); overweight/obese women had fewer hours of sleep than men and normal weight women (see Table 2).

## 4. Discussion

Adolescence is a time of transition from childhood to adulthood where many physiological and psychological changes occur that may affect the lifestyle and health of adolescents [27]. In accordance, some evidence shows that obesity and short sleep duration during childhood or adolescence may have important negative effects on physical and mental health, both frequently used as indicators of unhealthy lifestyle and poor health status [28,29]. Therefore, our aim was to estimate the prevalence of obesity in a sample of Mexican adolescents from the state of Tabasco in southern Mexico and evaluate, among other variables related to sleep and physical activities, the relationship between sleep duration per night and obesity by sex.

To begin, our first findings show that 47.6% of our total sample had overweight/obesity, which was higher in men (52.6% vs. 41.8%). Comparing this information with data previously reported, we could see a higher prevalence of overweight/obesity in this population; in 2012, it was 26.5% [7,8]. Moreover, in the 2016 Halfway National Health and Nutrition Survey, the prevalence of overweight/obesity was 39.2% in female adolescents and 33.5% in male adolescents [30].

There are multiple factors that could be involved in this higher prevalence; thus, it was necessary to also explore the participation of other variables that could be risk factors for overweight/obesity. In the present study, it could be observed that adolescents with overweight/obesity were younger than teens with normal weight (regardless of gender). Perhaps, this could be partially explained by a change in the election of food or nutritional transition [31]. Growing up, children, now adolescents, could have chosen foods with lower caloric intake [32]. However, future longitudinal studies should address this possibility as, in general, promotion of healthy food intake is generally not a focus of interest for adolescents. Therefore, it is necessary to promote the intake of healthier food in this population [33], where parents and school authorities may have an important role.

As another part of the analysis, we evaluated the association between hours of sleep and overweight/obesity. Previous studies found this association in the adult population [34], adolescent population [17,20,22,26,35,36], and children [37], and Mexico is no exception. We found that female adolescents with overweight/obesity were those with fewer sleep time hours.

In addition, we observed that women with normal weight slept 1.3 h more than men with normal weight. This result appears to be contradictory to some previous studies reported in the literature. Pengpid et al. [38], reported that women sleep fewer hours than men. However, it must be considered that most of the participants in that study were older than the participants included in our study. Alternatively, the study of Miguez, et al. [39] showed no differences in hours of sleep between men and women with similar ages to those reported herein. These discrepancies in the hours of sleep may be explained by the characteristics of the population, as well as the increased access to video games for men compared to women [40,41].

The mechanism underlying the association between sleep duration and obesity or even BMI remains poorly understood; however, there some explanations have been proposed. For example, findings of experimental studies have shown that sleep deprivation can affect food choices, leading to a reduction in the intake of vegetable and fruits and an increased intake of energy-dense foods, such as fast food or sugar-sweetened beverages [42,43]. To sum up, sleep loss has been proposed to be related to weight gain by increasing ghrelin levels and decreasing leptin levels, which stimulate appetite and the intake of excessive food [42,43]. In this way, it has been seen that, if children who sleep for a short duration spend more waking hours on sedentary activities, such as media use, they will have much more opportunities to ingest food or snacks [18,42]. The previously stated fact that, in our sample population, we found that females spend more time on the internet and men have more days of physical activities, is necessary to take into consideration in further studies. As sleep loss may have an effect on the energy expenditures, this could lead to fewer physical activities and more sedentary activities as subjects may feel more tired [42,43].

Some limitations can be identified in this study. This study was performed in Tabasco State and our results are not representative of the general Mexican population. Secondly, the characteristics associated with obesity were obtained from self-reports which could carry intrinsic respondent bias and measurement errors. Thirdly, only adolescent students were included in this survey; additionally, adolescents that were not enrolled in school during the survey were not included and this could have also biased the study. Lastly, metabolic parameters were not evaluated. In addition, sleeping time and other variables such as quality of sleep and awakening during the night, for example, were not considered, which may have an impact on metabolism alterations [44]; thus, these should be considered in future studies.

## 5. Conclusions

This study found an association between short hours of sleep and overweight/obesity in Mexican female adolescents. Similarly, adolescent women were found to spend more time on the internet, which might have contributed to the increase in overweight/obesity observed in this Mexican adolescent population. Strategies for the prevention of overweight/obesity should include a gender perspective to increase not only activity and exercise, but also increase the time of sleep in female adolescents.

## Figures and Tables

**Table 1:** Sleep variables, as well as sedentary and physical activities, among BMI groups according to sex.

Characteristics	Groups	BMI Normal Weight n = 452		BMI Overweight/Obese n = 411		Statistic
		*n*	*%*	*n*	*%*	*Cochran–Mantel–Haenszel*
Playing videogames	Men	120	63.5	145	67.1	χ^2^ = 0.2, *p* = 0.64
	Women	73	27.8	54	27.7	OR = 1.2
	Total	193	42.7	199	48.4	95% CI = 0.9–1.6
Watching TV daily	Men	169	89.4	203	94.0	χ^2^ = 0.01, *p* = 0.89
	Women	247	93.9	176	90.3	OR = 1.0
	Total	416	92.0	379	92.2	95% CI = 0.6–1.6
Internet use	Men	135	71.4	165	76.4	χ^2^ = 0.1, *p* = 0.69
	Women	196	74.5	141	72.3	OR = 1.0
	Total	331	73.2	306	74.5	95% CI = 0.7–1.4
Drinking water during night	Men	93	49.2	127	58.8	χ^2^ = 3.5, *p* = 0.06
	Women	123	46.8	99	50.8	OR = 1.4
	Total	216	47.8	226	55.0	95% CI = 0.9–2.1
Reading	Men	71	37.6	103	47.7	χ^2^ = 1.8, *p* = 0.17
	Women	132	50.2	98	50.3	OR = 1.1
	Total	203	44.9	210	51.1	95% CI = 0.8–1.5
Sports	Men	142	75.1	162	75.0	χ^2^ = 0.5, *p* = 0.47
	Women	165	62.7	83	42.6	OR = 0.9
	Total	307	67.9	274	66.7	95% CI = 0.7–1.2

Note: Numbers in bold show significant statistical difference.

**Table 2:** General linear models of the association between hours of sleep, as well as sedentary and physical activities, and obesity in Mexican adolescents.

Characteristics	Groups	BMI Normal Weight n = 452		BMI Overweight/Obese n = 411		Statistic
		*Mean*	*S.D.*	*Mean*	*S.D.*	*General Linear Model*
Age	Men	13.0	0.9	12.2	0.7	**BMI F = 9.8, *p* = 0.003**
	Woman	13.4	1.0	12.6	0.9	Sex F = 1.9, *p* = 0.17
	Total	13.2	0.9	12.3	0.8	BMI × sex F = 0.002, *p* = 0.96
Hours of sleep	Men	8.3	0.7	8.6	1.3	BMI F = 3.6, *p* = 0.06
	Woman	9.6	1.6	7.9	1.3	Sex F = 0.8, *p* = 0.35
	Total	8.9	1.6	8.4	1.3	**BMI × sex F = 7.6, *p* = 0.008**
Videogames Hours/day	Men	1.9	1.0	1.9	0.9	BMI F = 1.3, *p* = 0.24
	Woman	2.3	1.2	1.6	1.1	Sex F = 0.02, *p* = 0.87
	Total	2.1	1.1	1.8	0.9	BMI × sex F = 1.5, *p* = 0.22
Videogames Days/week	Men	4.2	1.9	4.2	2.1	BMI F = 0, *p* = 0.99
	Woman	3.7	2.5	3.7	2.7	Sex F = 0.6, *p* = 0.42
	Total	4.0	2.2	4.1	2.2	BMI × sex F = 0.002, *p* = 0.96
Watching TV Hours/day	Men	2.0	0.7	1.9	0.6	**BMI F = 129.4, *p* < 0.001**
	Woman	2.1	0.7	2.0	0.5	Sex F = 0.1, *p* = 0.74
	Total	2.1	0.7	1.9	0.6	BMI × sex F = 0.006, *p* = 0.94
Internet use Hours p/day	Men	2.2	1.7	2.0	1.2	BMI F = 1.1, *p* = 0.28
	Woman	2.4	1.5	3.7	3.1	**Sex F = 4.0, *p* = 0.05**
	Total	2.3	1.6	2.4	2.0	BMI × sex F = 2.1, *p* = 0.14
Internet use Days/week	Men	4.6	2.4	5.2	2.4	BMI F = 0.5, *p* = 0.47
	Woman	5.4	2.0	5.7	2.1	Sex F = 0.8, *p* = 0.35
	Total	5.0	2.2	5.4	2.3	BMI × sex F = 0.05, *p* = 0.82
Reading Hours/day	Men	1.1	0.5	1.4	1.0	BMI F = 0.04, *p* = 0.83
	Woman	1.5	0.8	1.3	0.5	Sex F = 0.6, *p* = 0.41
	Total	1.3	0.7	1.4	0.9	BMI × sex F = 1.1, *p* = 0.29
Sports Hours/day	Men	2.4	1.3	2.6	1.4	BMI F = 0.2, *p* = 0.62
	Woman	2.0	1.0	2.1	1.2	Sex F = 1.9, *p* = 0.17
	Total	2.2	1.2	2.5	1.3	BMI × sex F = 0.01, *p* = 0.91
Sports Days/week Sports	Men	4.7	2.0	3.6	2.1	BMI F = 0.5, *p* = 0.46
	Woman	2.8	2.3	3.1	1.1	**Sex F = 4.2, *p* = 0.04**
	Total	3.8	2.3	3.4	1.9	BMI × sex F = 1.4, *p* = 0.23

Note: Numbers in bold show significant statistical difference.

## Data Availability

Data is available upon request.
